# Osteopathic Manipulative Treatment as an Adjunctive Therapy for Post-mastectomy Pain Syndrome: A Case Report

**DOI:** 10.7759/cureus.114567

**Published:** 2026-08-15

**Authors:** Sahana Natesan, Grace Young, Monica Khattak, Korrie Beverley-Waters, Kristen Conrad-Schnetz

**Affiliations:** 1 General Surgery, Touro College of Osteopathic Medicine, Middletown, USA; 2 General Surgery, Cleveland Clinic South Pointe Hospital, Cleveland, USA; 3 Osteopathic Medicine, Cleveland Clinic South Pointe Hospital, Cleveland, USA

**Keywords:** breast cancer survivorship, osteopathic manipulative therapy, post-mastectomy pain syndrome, refractory cancer pain, triple-negative breast carcinoma

## Abstract

Post-mastectomy pain syndrome (PMPS) is a common yet often under-treated complication of breast cancer therapy, affecting an estimated 20-60% of patients. Characterized by persistent pain lasting greater than three months following breast surgery, PMPS is multifactorial in etiology, involving neuropathic injury, post-surgical fibrosis, radiation-induced tissue changes, and musculoskeletal dysfunction. Current management strategies emphasize a multidisciplinary approach, including pharmacological therapies and non-pharmacological modalities. Despite this, treatment outcomes remain variable, and many patients continue to experience refractory symptoms. Osteopathic manipulative treatment (OMT), a hands-on approach targeting somatic dysfunction and improving lymphatic and musculoskeletal function, represents a potentially valuable yet underexplored adjunct in this population. We present the case of a 72-year-old female with stage IIIA triple-negative breast cancer who developed refractory PMPS following mastectomy, axillary dissection, and radiation. After limited improvement with massage therapy, she underwent OMT incorporating myofascial release, muscle energy, and balanced ligamentous tension. She reported improvement in pain and functional mobility, with decreased reliance on adjunctive therapies. This case highlights OMT as a promising adjunct for PMPS warranting further investigation.

## Introduction

Post-mastectomy pain syndrome (PMPS) is a well-recognized, yet often under-treated, complication of breast cancer therapy. PMPS typically presents as persistent pain involving the chest wall, shoulder, or axillary region following surgical intervention to the breast [1]. Studies have shown that PMPS affects an estimated 20-60% of patients [2,3]. Studies have shown that PMPS can significantly impair functional recovery and quality of life [4]. The pathophysiology of PMPS is multifactorial, involving neuropathic injury (most notably to the intercostobrachial nerve) [5,6], as well as contributions from post-surgical fibrosis, radiation-induced tissue changes, and musculoskeletal dysfunction [7].

Current management strategies emphasize a multidisciplinary approach [8,9], incorporating pharmacological therapies, such as antidepressants [10] and nerve blocks [11], as well as non-pharmacological modalities, including physical therapy, massage therapy, and music therapy [12-14]. However, treatment outcomes remain variable, and many patients continue to experience persistent symptoms despite these interventions. Osteopathic manipulative treatment (OMT), a hands-on approach aimed at addressing somatic dysfunction and improving lymphatic and musculoskeletal function, represents a potentially valuable yet underexplored adjunct in this population [15,16]. Here, we present a case of PMPS in a patient with triple-negative breast cancer who demonstrated symptomatic improvement following OMT, highlighting its potential role as an adjunctive therapy in post-mastectomy survivorship care.

## Case presentation

**Figure 1:**
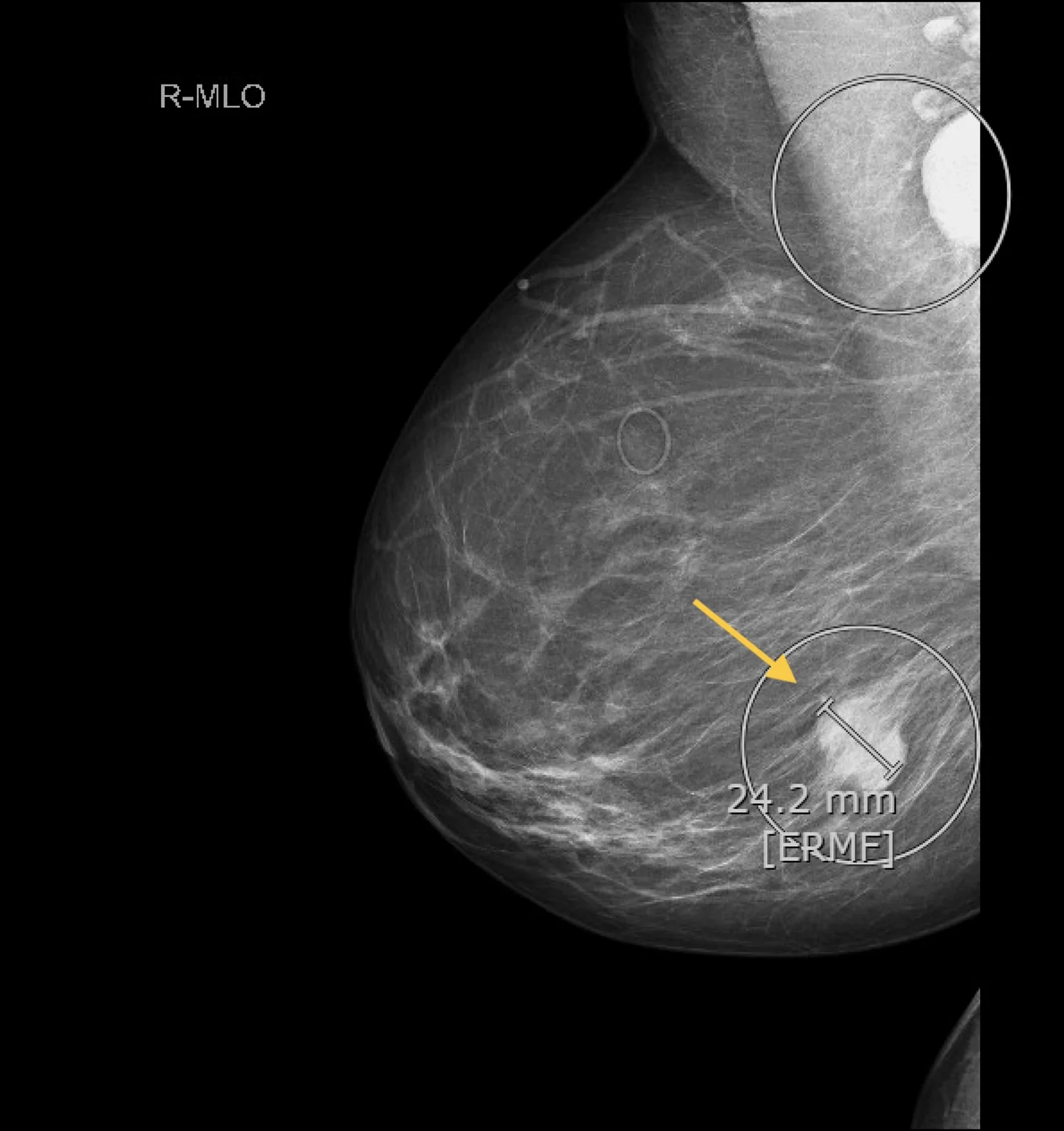
Screening mammogram demonstrating a focal asymmetry in the right breast (arrow)

**Figure 2:**
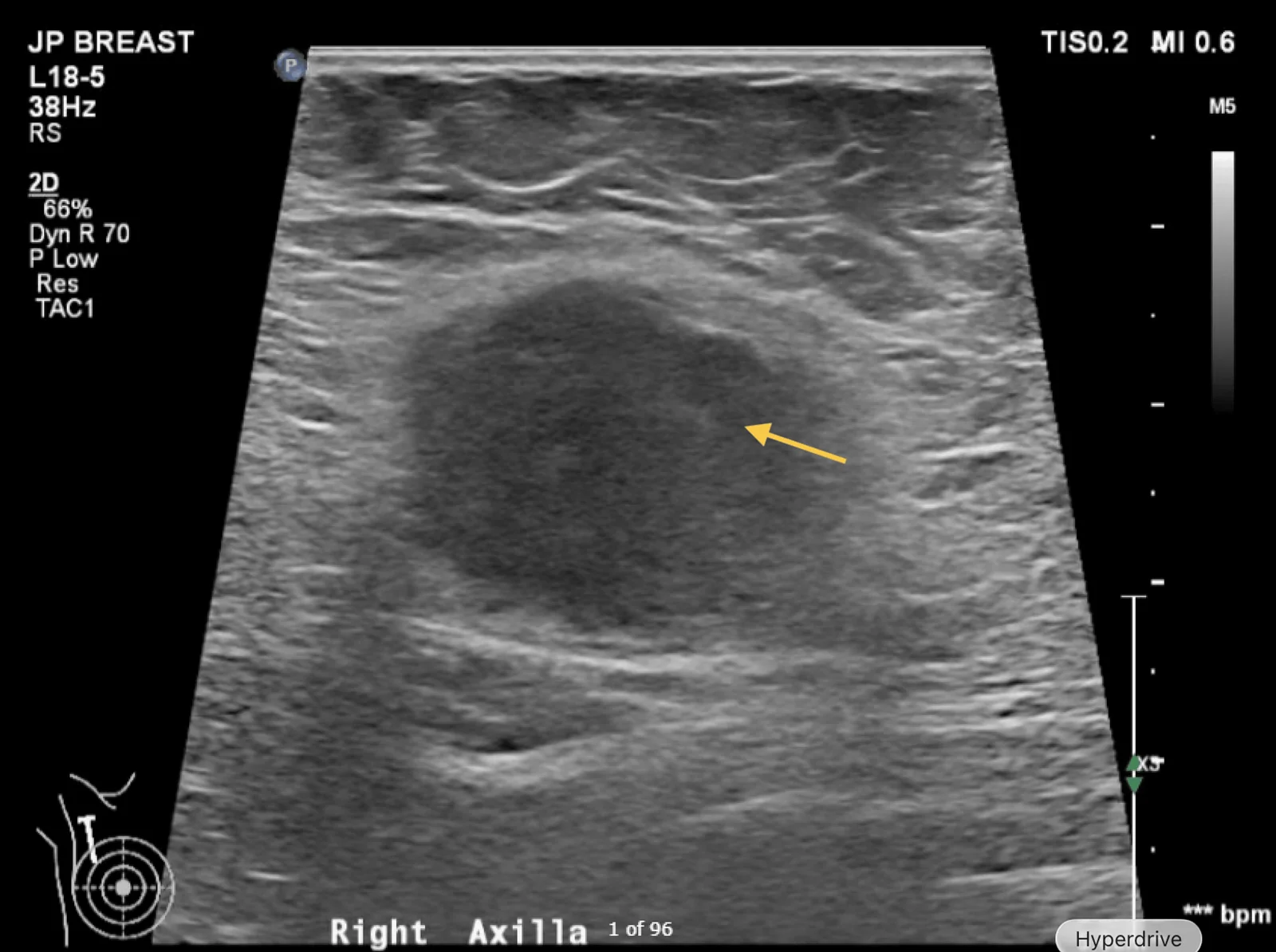
Targeted ultrasound demonstrating a suspicious right axillary lesion (arrow).

A 72-year-old postmenopausal African American female was diagnosed with right breast invasive ductal carcinoma following an abnormal screening mammogram in the fall of 2020. Screening mammography demonstrated a 2.5-cm focal asymmetry in the right breast (Figure 1), and subsequent targeted ultrasound identified a suspicious right axillary lesion (Figure 2). Ultrasound-guided biopsy of both sites confirmed invasive ductal carcinoma with solid-papillary features.

Immunohistochemical analysis revealed triple-negative breast cancer (estrogen receptor (ER)-negative, progesterone receptor (PR)-negative, and HER-2 negative disease). On clinical examination, a 4 cm mobile mass was palpated in the right breast at the 7 o’clock position, along with a 5 cm mobile axillary mass adjacent to the pectoralis major, corresponding to nodal involvement. No abnormalities were identified in the contralateral breast or axilla. The patient was staged as clinical stage IIIA (T2N2).

She underwent neoadjuvant chemotherapy followed by right modified radical mastectomy with axillary lymph node dissection. Final surgical pathology demonstrated a complete pathologic response, with no residual carcinoma identified in the breast or axillary lymph nodes (ypT0N0). Given her initial presentation with triple-negative disease and significant nodal involvement, she subsequently received adjuvant external beam radiation therapy to the right chest wall and axilla (total dose 5000 cGy over 32 fractions), which she tolerated well.

Immediately following mastectomy, the patient developed persistent right-sided chest wall pain. She described the pain as tight, burning, and intermittently sharp and shooting, localized to the mastectomy site with associated decreased range of motion of the right upper extremity. Symptoms persisted for more than one year despite multiple courses of physical therapy, occupational therapy, breast rehabilitation, breast psychology, and massage therapy, with only minimal relief. Evaluation with chest radiography and right shoulder/upper extremity radiographs demonstrated no acute abnormalities aside from degenerative spinal changes. Given the chronic postoperative course, characteristic symptom distribution, and exclusion of alternative causes, a diagnosis of post-mastectomy pain syndrome was made.

Osteopathic structural examination revealed notable somatic dysfunction, including tissue texture changes of the right anterior chest wall, restricted right upper extremity abduction secondary to pain, increased paraspinal hypertonicity, and decreased scapular mobility. The patient was referred to Osteopathic Neuromusculoskeletal Medicine (ONMM) for further management. She subsequently underwent monthly OMT sessions for approximately one year, followed by maintenance treatments every three to four months as needed. Treatment targeted the chest wall, thoracic spine, cervical spine, and surrounding musculature using myofascial release, muscle energy, balanced ligamentous tension, and percussion hammer techniques. Following initiation of OMT, the patient reported improvement in pain, lymphatic congestion, and functional mobility, with decreased reliance on massage and physical therapy.

## Discussion

PMPS remains a challenging sequela of breast cancer treatment, particularly in patients with combined surgical and radiation exposure [9,17,18]. This case underscores the persistence of symptoms despite conventional therapies and highlights the potential role for osteopathic manipulative treatment (OMT) in addressing refractory pain and improving functional outcomes.

PMPS is increasingly understood as a multifactorial condition involving not only neuropathic injury but also soft tissue fibrosis and biomechanical dysfunction [7,9,19]. In this case, structural findings, including anterior chest wall restriction, scapular dyskinesia, and paraspinal hypertonicity, suggest that musculoskeletal and fascial components significantly contributed to the patient’s symptoms [19-21]. Such factors are often incompletely addressed by standard pharmacologic or interventional approaches.

OMT provides a targeted approach to these structural abnormalities. Techniques such as myofascial release, muscle energy, and balanced ligamentous tension aim to improve tissue mobility, restore joint mechanics, and reduce compensatory strain patterns [13,22]. OMT has also been shown to enhance lymphatic drainage and modulate autonomic tone. These techniques become particularly relevant in the setting of post-surgical and radiation-induced changes [23,24]. This patient’s improvement in pain, range of motion, and reduced reliance on adjunctive therapies following OMT suggests a meaningful therapeutic contribution.

Existing evidence for osteopathic and manual approaches to cancer-related pain, while limited, is broadly consistent with the improvement observed in this case. In a randomized placebo-controlled trial, Galeazzi et al. found that OMT reduced pain in palliative care patients, while Arienti et al. reported improvements in pain and quality of life among geriatric oncology patients receiving OMT [15,16]. A systematic review and meta-analysis by Pinheiro da Silva et al. similarly supported manual therapy for chronic musculoskeletal pain in female breast cancer survivors, although PMPS was not examined separately [13]. Most directly, Goyal et al. described a case of post-mastectomy lymphedema in which OMT was associated with improvements in pain, upper-limb disability, and lymphatic volume [25]. The present case adds to the literature by describing the use of OMT in a patient with refractory PMPS.

This case has inherent limitations, including the absence of standardized pain outcome measures and baseline pain severity scores, the lack of a control condition, and the patient's prior use of massage therapy. The subjective nature of the reported improvement also introduces the possibility of expectation or placebo effects associated with hands-on treatment. As such, a causal relationship between OMT and the patient's improvement cannot be established. Nevertheless, the patient’s reported improvement in pain and functional mobility, along with decreased need for additional adjunctive therapies, suggests a potential benefit of manual therapy, particularly OMT, for cancer-related pain.

While this case adds to the growing literature supporting manual therapy for cancer-related pain, future prospective and randomized controlled studies should incorporate standardized pain measures, objective range-of-motion or functional assessments, and clearly defined OMT treatment protocols to permit reproducible evaluation of clinical outcomes.

## Conclusions

This case describes subjective improvement in pain and functional mobility following the addition of OMT to multimodal rehabilitation in a patient with persistent PMPS. OMT represents a valuable adjunctive therapy for selected patients with PMPS and warrants further study.
